# ALK is frequently phosphorylated in Merkel cell carcinoma and associates with longer survival

**DOI:** 10.1371/journal.pone.0252099

**Published:** 2021-05-24

**Authors:** Jenni Jaatinen, Tuukka Veija, Marko Salmikangas, Tom Böhling, Harri Sihto, Virve Koljonen

**Affiliations:** 1 Department of Pathology, University of Helsinki, Helsinki, Finland; 2 Department of Plastic Surgery, University of Helsinki and Helsinki University Hospital, Helsinki, Finland; Universita degli Studi di Milano-Bicocca, ITALY

## Abstract

Merkel cell carcinoma (MCC) is a rare skin cancer with only limited therapeutic options for advanced disease. We previously showed that oncogene ALK is frequently expressed at the RNA level in MCC and further that ALK positivity by immunohistochemistry is frequent and correlates strongly with Merkel cell polyomavirus (MCPyV) positivity. In this study, we investigated whether ALK receptor is active in MCC tumor samples and MCC cell lines, and whether ALK would be a prospective treatment target in MCC. We utilized tissue microarrays constructed from 136 primary MCC tumor samples as well as nine previously established MCC cell lines to determine the presence of ALK and phosphorylated ALK (p-ALK) via immunohistochemistry. Almost half of the analyzed MCC tumors displayed ALK phosphorylation (47.8%). Analysis of MCC tumor samples revealed that the presence of p-ALK correlated to MCPyV positivity, younger age, nonexistence of metastases at diagnosis and ultimately to better MCC-specific survival. In contrast to MCC tumor samples only two out of nine MCC cell lines showed only low ALK phosphorylation by immunohistochemistry. Our study reveals clear disparity in ALK activity between patient derived tumors and cell line samples and therefore, more advanced disease models such as xenografts are necessary to resolve whether ALK is a useful treatment target in MCC.

## Introduction

Merkel cell carcinoma (MCC) is a rare and aggressive cutaneous tumor with disputed origin, divided into two groups: Merkel cell polyomavirus (MCPyV) positive and MCPyV-negative tumors. [1–3] For local MCC, surgical excision is typically sufficient treatment, however different treatment modalities are needed for advanced MCC. While new immunotherapy regimens, such as pembrolizumab and avelumab have been efficient in treating metastasized disease, they are ineffective against some MCC tumors and currently there is no adequate tool to identify the cases that could benefit from immunotherapy [4,5].

Anaplastic lymphoma kinase (ALK) is a tyrosine kinase receptor that normally regulates the development of the nervous system during embryogenesis, although it can entail oncogenic properties. After the receptor activation through phosphorylation of its specific tyrosine residues, the phosphorylated ALK (p-ALK) activates multiple important cell cycle regulating cascades such as the mitogen activated protein kinase (MAPK) and extracellular signal regulated kinase (ERK) pathway and phosphoinositide 3-kinase (PI3K) and protein kinase B (PBK/AKT) pathway [6]. Originally known for its role in the oncogenic fusion gene in anaplastic large cell lymphoma, ALK has been established as a treatment target in various cancers, including subgroups of non-small cell lung cancer and colorectal carcinoma [7–10].

Frequent expression of oncoprotein ALK in MCC by immunohistochemistry (IHC) was first reported in 2013 by Filtenborg-Barnkob et al. [11] In our previous studies, we investigated the gene expression patterns in MCC for new potential therapeutic targets. We found that ALK is frequently expressed at the RNA level in MCC regardless of the MCPyV status [12]. Furthermore, we validated that ALK expression by IHC is frequent, and 51% of the tumours exhibit strong staining for ALK and a positive IHC staining clearly correlates to MCPyV positivity [13].

Based on our previous findings, we sought to further elucidate the suitability of ALK as a therapeutic target for MCC treatment. Here, we investigated whether ALK is phosphorylated in MCC primary tumor samples and nine previously established MCC cell lines, and whether p-ALK status correlates with patient characteristics including MCC-specific and overall survival.

## Results

### Patient cohort

MCC patients’ relevant clinical data are summarized in Table 1. Our cohort consisted of 97 (71.3%) women and 39 (28.7%) men, whose age at the time of diagnosis ranged from 27 to 100 years old (median, 79 years). The primary tumor was most often located in the head and neck region (n = 70, 51.9%) followed by limbs (n = 48, 35.6%) and trunk (n = 17, 12.6%), respectively. Distant metastases were present at the time of diagnosis in 17 (12.5%) subjects. The tumor size ranged from 6 to 85 mm in diameter (median 15.5 mm). The stage at diagnosis was unavailable for 33 cases due to missing information regarding tumor size or the lymph node status at the time of diagnosis. The clinical follow up ended March 2019 and the average follow up time was 5,6 years (range 0,02–33,2 years).

**Table 1:** The demographic data of 136 MCC patients with ALK and p-ALK immunostaining results.

Number of patients	136
**Sex**	
Male, n (%)	39 (28.7%)
Female, n (%)	97 (71.3%)
**Age at diagnosis**	
Mean, years	76.85
Range, years	27–100
**Location of the primary tumor**	
Head and neck, n (%)	70 (51.9%)
Upper extremity, n (%)	28 (20.7%)
Lower extremity, n (%)	20 (14.8%)
Torso, n (%)	17 (12.6%)
NA	1
**MCPyV status**	
Positive, n (%)	84 (62.2%)
Negative, n (%)	51 (37.8%)
NA	1
**Stage at diagnosis**	
I	51 (49,5%)
IIa, IIb	32 (31,1%)
III	12 (11,7%)
IV	8 (7,7%)
NA	33
**Survival by 3/2019**	
Alive	24 (17.6%)
MCC specific death	37 (27.2%)
Death to other cause	75 (55.2%)

NA not available.

### Tumor sample analysis

**Fig 1:**
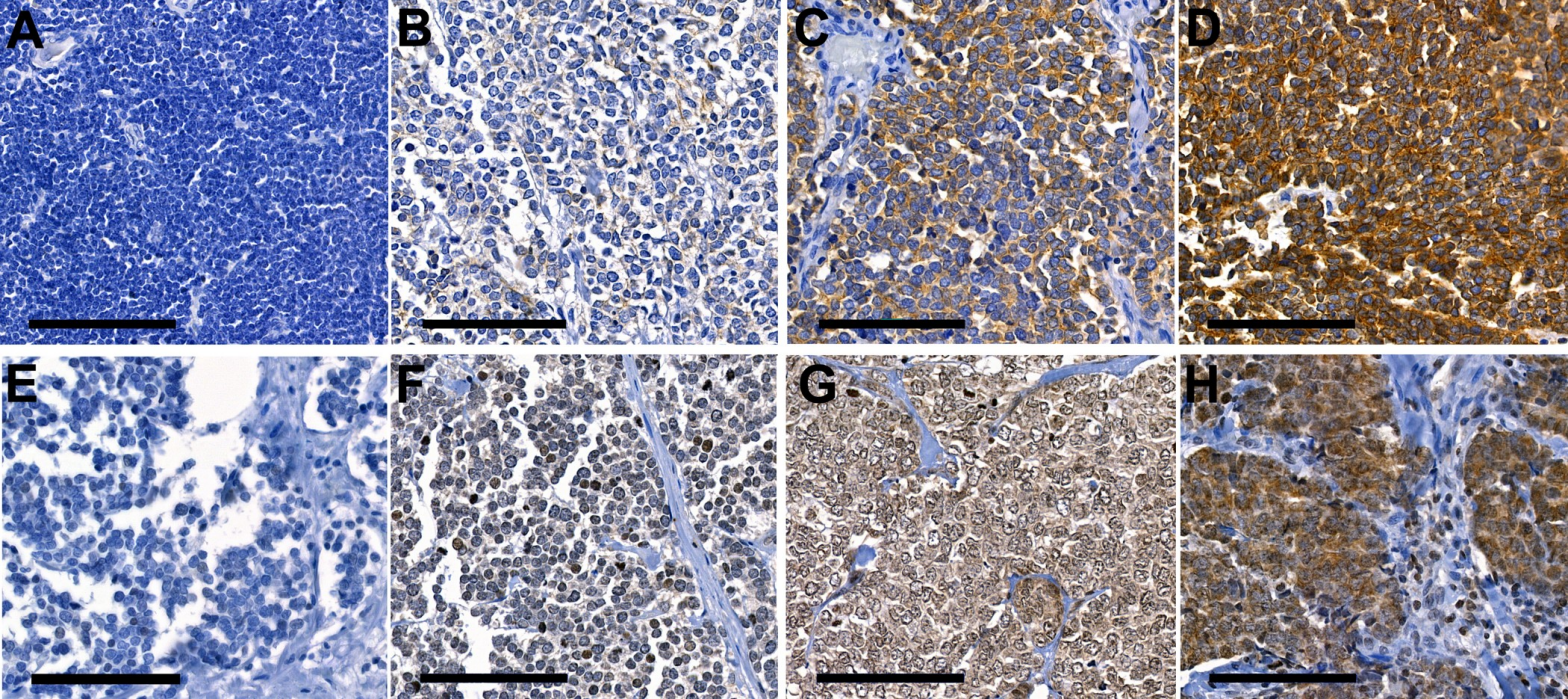
The grading of Immunohistochemistry staining as negative, low, intermediate or high is displayed for ALK (A to D) and p-ALK (E to H) across MCC TMA samples. 40x Magnification. Size bar 100μm.

The tumor sample IHC staining patterns are demonstrated in Fig 1. ALK IHC was positive in 101 (74.3%) out of 136 MCCs (negative in 35, low in 23, intermediate in 38 and high in 40 tissue samples). p-ALK was detected in 65 (47.8%) samples (negative in 71, low in 35, intermediate in 24 and high in 6 samples). Positivity for ALK and p-ALK was frequently detected in the same tumor samples (*P* < 0.001). A total of 54 (39.7%) of 136 tumors expressed both markers, whereas 24 tumors (17.6%) expressed neither. (Table 2) Due to a small sample size of some factors, in statistical analyses groups were combined for the cross-tabulation analysis. Thus, samples with negative or low ALK expression or ALK phosphorylation were combined. In addition, samples with intermediate or high ALK expression or phosphorylation were combined into a separate group.

**Table 2:** ALK and p-ALK expression by IHC in MCC TMAs representing 136 primary tumors.

		p-ALK expression			
		Negative	Low	Intermediate	High
**ALK expression**	Negative	24 (17.6)	9 (6.6)	2 (1.5)	-
	Low	16 (11.7)	6 (4.4)	1 (0.7)	-
	Intermediate	20 (14.7)	8 (5.8)	8 (5.8)	2 (1.4)
	High	11 (8)	12 (8.8)	13 (9.5)	4 (2.9)

Number of cases in each category and percentages of all cases are presented in parenthesis.

ALK and p-ALK positivity had strong association with the MCPyV large T-antigen (LT) expression (*P* < 0.001 for ALK and P = 0.032 for p-ALK). In addition, high and intermediate p-ALK positive cases had 5.5 years lower median age at diagnosis compared to low- and negative cases (*P* = 0.005). Furthermore, the presence of p-ALK correlated with an absence of metastatic disease at the time of diagnosis (*P* = 0.012). All the metastases (n = 17) were found in tumors that had either negative or low ALK phosphorylation.

**Fig 2:**
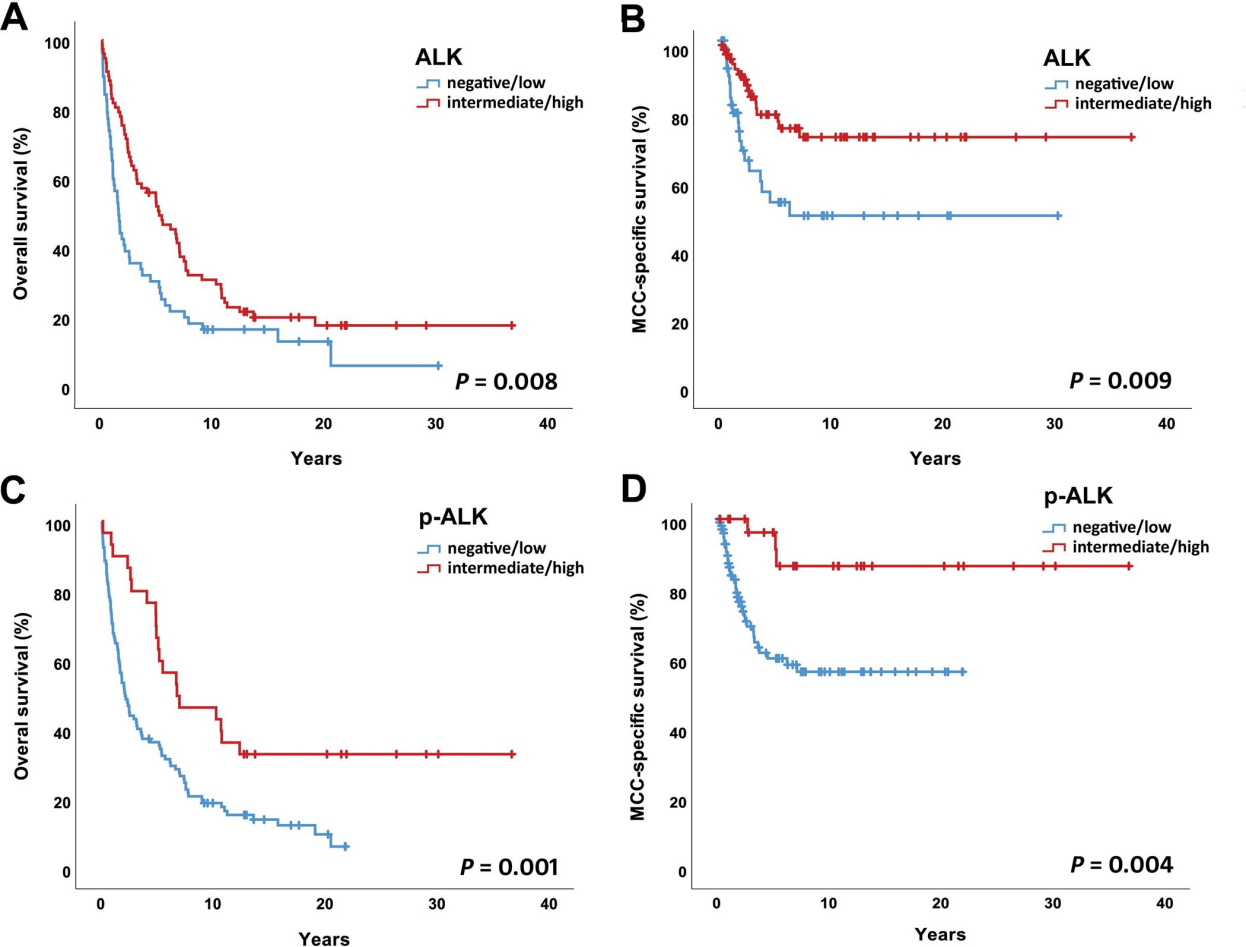
Overall and MCC-specific survival in relation to ALK and p-ALK status by immunohistochemistry.

Overall survival and MCC-specific survival curves according to the ALK and p-ALK status are presented in Fig 2. ALK positivity as well as p-ALK positivity correlated with better overall and MCC specific survival. The median 5-year overall survival rates for ALK negative, low, intermediate and high expressing tumors were 25.7%, 26.1%, 44.7% and 49.7% (*P* = 0.061), respectively. The corresponding MCC-specific 5-year survival rates were 51.1, 56.7, 68.0 and 82.4 percent (*P* = 0.034). Regarding p-ALK status, the median 5-year overall survival rates were 27.9 (negative), 42.9 (low), 50.0 (intermediate) and 83.3 (high) percent (*P* = 0.004) respectively. Corresponding MCC-specific median 5-year survival rates were 58.1, 64.3, 81.4 and 100 percent (*P* = 0.027).

Due to association of MCPyV LT and p-ALK positivity, we examined whether ALK or p-ALK expression is independently associated with survival. Younger than median age at the time of diagnosis (79 years), stage at diagnosis, LT expression and intermediate to high ALK and p-ALK were entered as a covariables to the Cox multivariable proportional hazards models. In addition to the lower stage, both ALK and p-ALK positivity were independent prognostic factors for a better MCC-specific outcome. p-ALK positivity had more impact on MCC-specific survival compared to ALK positivity (HR 0.25, 95% CI 0.068–0.87 and *P* = 0.032 for p-ALK and HR 0.39, 95% CI 0.17–0.88 and *P* = 0.023 for ALK). In the overall survival analyses, the younger age, lower stage, LT expression and ALK expression (HR 0.49, 95% CI 0.30–0.79 and *P* = 0.004) were independent prognostic factors, whereas p-ALK expression was not. Tables 3 and 4 present the results of the multivariable analyses on MCC specific and overall survival.

**Table 3:** Multivariable Cox hazards analysis on MCC-specific survival.

Covariate	β(SE)	HR of death (95% CI)	*P* value
**Stage at diagnosis**			
**I**		1.00	
**IIa, IIb**	0.286 (0.600)	1.33 (0.41 to 4.3)	0.634
**III**	2.437 (0.507)	11.44 (4.24 to 30.91)	<0.0001
**IV**	2.135 (0.666)	8.46 (2.29 to 31.24)	0.001
**Age less than median at diagnosis**	0.023(0.015)	1.02 (0.99 to 1.06)	0.126
**MCPyV LT expression in tumor cells**			
**Present vs. absent**	-0.1085 (0.440)	0.338 (0.14 to 0.80)	0.014
**ALK phosphorylation by IHC**			
**Intermediate or high vs. Negative or low**	-1.404 (0.654)	0.25 (0.068 to 0.87)	0.032

β = regression coefficient of hazards function; SE = standard error; HR = hazard ratio; CI = confidence interval.

**Table 4:** Multivariable Cox hazards analysis on overall survival.

Covariate	β(SE)	HR of death (95% CI)	*P* value
**Stage at diagnosis**			
**I**		1.00	
**IIa, IIb**	0.250 (0.272)	1.28 (0.75 to 2.19)	0.359
**III**	1.254 (0.349)	3.51 (1.77 to 6.95)	<0.001
**IV**	1.275 (0.442)	3.58 (1.51 to 8.51)	0.003
**Age less than median at diagnosis**	0.058 (0.011)	1.06 (1.04 to 1.08)	<0.001
**MCPyV LT expression in tumor cells**			
**Present vs. absent**	-0.765 (0.252)	0.465 (0.43 to 1.01)	0.002
**ALK phosphorylation by IHC**			
**Intermediate or high vs. Negative or low**	-0.513 (0.289)	0.60 (0.34 to 1.05)	0.075

β = regression coefficient of hazards function; SE = standard error; HR = hazard ratio; CI = confidence interval.

### Cell line analysis

**Fig 3:**
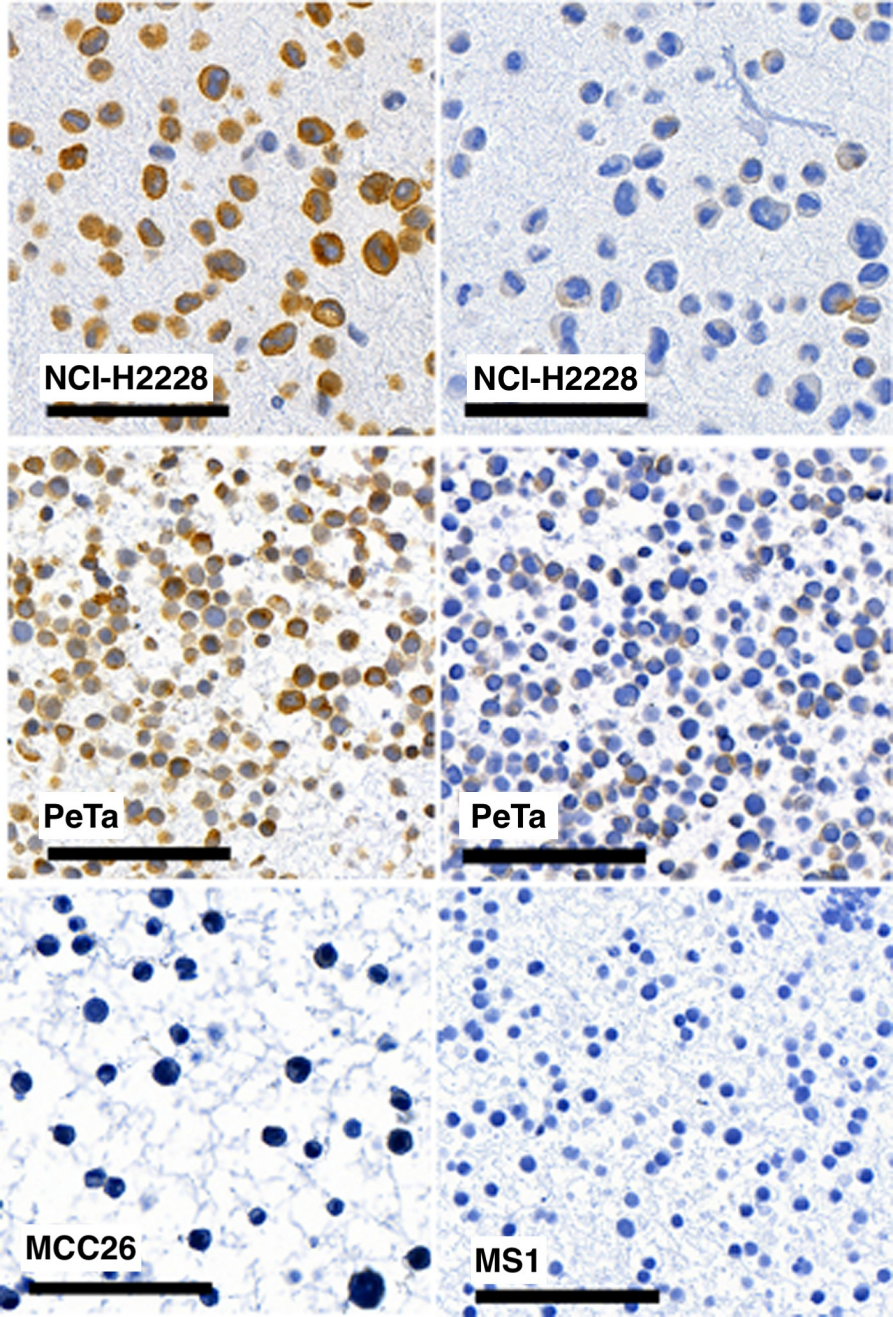
Analysis of the IHC expression of ALK (left column) and p-ALK (right column) in lung cancer cell line NCI-H2228 and in MCC cell lines PeTa and MCC26/MS1. Size bar 100μm.”.

ALK and p-ALK status were investigated in the nine established MCC cell lines and in the lung carcinoma cell line NCI-H2228, which harbors a fusion gene formed from genes Echinoderm microtubule-associated protein-like 4 (*EML4*) and *ALK*, and was used as a positive control. By IHC, ALK expression was detected in five MCPyV-positive MCC cell lines (MS1, MKL1, MKL2, PeTa and WaGa, respectively) and three MCPyV-negative cell lines (UISO, MCC13 and MCC14/2). Only the MCC26 cell line was ALK-negative. (Table 5). Analysis of p-ALK with the same method revealed only low positive staining in MCC13 and PeTa cells. Representative images of ALK and p-ALK staining in selected MCC cell lines are displayed in Fig 3.

**Table 5:** Summary of ALK and p-ALK status in MCC cell lines and positive control cell line (NCI-H2228) by IHC.

Cell line	ALK positivity	p-ALK positivity	MCPyV positivity
**UISO**	**++**	-	-
MCC13	+	+	-
MCC14/2	+	-	-
MCC26	-	-	-
MS1	+	-	+
MKL1	++	-	+
MKL2	++	-	+
PETA	++	+	+
WAGA	++	-	+
NCI-H2228	++	+	NA

“+” = low expression, “++” = high expression and “-” = negative expression.

## Discussion

In this study we aimed to determine whether the ALK receptor is active in MCC to learn if ALK could serve as a potential therapeutic target. Therefore, we analyzed ALK phosphorylation by IHC in patient derived MCC samples as well as in previously established MCC cell lines. We demonstrated the presence of p-ALK by IHC in almost half of the tumor samples included in our cohort (47.8%). Both the expression of total ALK and the presence of p-ALK correlated with tumor MCPyV positivity and, further, to a better survival. Interestingly, p-ALK positivity was associated with a younger age as well as nonexistence of metastases at diagnosis. Especially the six subjects with high p-ALK positivity had a 100% 5-year MCC-specific survival compared to only 58.1% survival in p-ALK negative cases (71 subjects). The association of tumor MCPyV positivity and better prognosis was established in multiple previous studies [3,14,15]. On the other hand, we previously observed a relationship between ALK and MCPyV [13]. Here, we showed that intermediate to high p-ALK positivity was an independent prognostic factor for MCC-specific survival.

A high expression of ALK in MCC was previously documented both at protein and RNA level. However, despite in-depth analysis, no activating ALK mutations or fusion genes involving ALK have been discovered [11,12]. It is known that in virus related cancers, viral oncoproteins can regulate the expression of key oncogenes and tumor suppressor genes. One example is Human Papilloma Virus (HPV) oncoproteins E6 and E7 that can regulate various tumor suppressors and oncoproteins in HPV associated lung carcinoma [16]. It is possible that in MCC, MCPyV oncoproteins facilitate the activation of ALK.

A high ALK expression without an activating mutation or fusion of the *ALK* gene is also observed in Hepatocellular carcinoma (HCC) and it is correlated to presence of Hepatitis C Virus infection and further to earlier development of metastasis and lower survival [10]. In addition to HCC, ALK overexpression or phosphorylation is linked to unfavorable course of disease also in inflammatory breast cancer [17] as well as neuroblastoma, where ALK phosphorylation is linked to activating mutations of the *ALK* gene [18]. The positive effect of ALK inhibition in HCC *in vitro* was recently demonstrated by Yu *et al*. Two approved inhibitors crizotinib and ceritinib suppressed the proliferation of HCC cell lines and inhibited the ALK phosphorylation; moreover, ceritinib treatment induced cell apoptosis [19]. Therefore, it seems that in HCC ALK inhibition is beneficial, even though the mechanism of ALK activation is still unknown.

Overexpression of ALK is also reported in basal cell carcinoma (BCC) of the skin. Ning *et al*. showed that ALK is frequently phosphorylated as well as in BCC but also in human keratinocytes and that ALK inhibitor crizotinib inhibited keratinocyte proliferation *in vitro*. The authors suggested that ALK could be a therapeutic target in BCC, however the lack of commercial BCC cell lines hinders further investigation [20].

To our knowledge this is the first study exploring ALK expression and phosphorylation in the MCC cell lines. The major question rising due to our study is why majority of the MCC cell lines lack ALK phosphorylation although patient derived tumor samples display p-ALK positivity by IHC. We hypothesize that the *in vivo* MCC tumor microenvironment could promote activation of ALK via an unknown mechanism. However, the cell line models lack the tumor microenvironment that consist of different cell types surrounding the cancer cells such as epithelial cells, fibroblasts, lymphocytes etc, lymphovascular network and extracellular matrix, as well as all the immanent cytokines, growth factors and other molecules. The tumor microenvironment has been widely under scope in recent years and it seems to have a central role in cancer treatment response [21]. In MCC, the function and key factors of the tumor microenvironment are quite poorly understood. Previous literature is yet indecisive on what are the actual ligands binding and activating the ALK receptor, but molecules such as Midkine (MDK) and heparin have been suggested [22]. Keeping in mind the correlation of MCPyV positivity and expression of ALK and p-ALK, ALK phosphorylation may be facilitated by the virus or by some other unknown factor present in the cells or by the tumor microenvironment.

Although we detected ALK phosphorylation by IHC in almost half of the MCC tumors studied, it is still infeasible to make conclusions about the potential *in vivo* efficacy of ALK inhibitors in MCC tumors. Even tough expression of ALK and p-ALK is strongly correlated and others have reported similar findings regarding ALK expression in MCC tumor samples, it is possible that the differences between cell lines and the tumor data might be due to antibody cross-reactions. We acknowledge the limitation in this study, that IHC is done with single antibodies for ALK and p-ALK. However, the antibodies we applied here are widely used in routine; Total ALK antibody (D5F3) was well validated in previous studies not only for MCC [11,13] but also for other cancers such as non-small-cell lung carcinoma [23]. p-ALK IHC was done with antibody clone Tyr1604 whose manufacturer declares that the clone detects ALK only when phosphorylated at Tyr1604, which is equivalent to Tyr664 of NPM-ALK fusion protein, and that it may cross-react with other activated protein tyrosine kinases including EGFR. Based on our previous studies, MCC tumors lack EGFR expression at RNA level and only a small proportion of MCPyV-negative tumors showed EGFR positivity by IHC [12,13]. Therefore, it would be unlikely that the observed ALK phosphorylation in this study would have been due to antibody cross reaction with EGFR. Furthermore, antibody Tyr1604 has been utilized frequently to detect p-ALK in multiple malignancies including HCC, non-small cell lung carcinoma as well as neuroblastoma [19,24–26]. Thus, we suggest that our observation of ALK expression and phosphorylation in MCC tumor samples is genuine.

Since the majority of established MCC cell lines lack ALK phosphorylation, the next approach would be to study ALK phosphorylation in patient derived MCC xenograft models. Ultimately, *in vitro* drug testing with xenograft models or even *in vivo* trials could be necessary in order to resolve whether ALK inhibitors would be beneficial in treating MCC.

## Materials and methods

The study plan and its protocol were approved by the Ethics Committee of Helsinki University Hospital and the local review board (Project identification code HUS/221/2017). The Ministry of Health and Social Affairs granted us the permission to collect patient data and the National Supervisory Authority for Welfare and Health (VALVIRA) (Code: 4942/05.01.00.06/2009) allowed us to collect and analyze the tissue samples. The need for a written informed consent was waived by the Ethics Committee.

### Patients, clinical data, and tissue samples

Data on patients diagnosed with MCC in Finland were obtained from the Finnish Cancer Registry and Helsinki University Hospital patient files. Clinical details were extracted from hospital records in between 2013 and March 2019. Patients selected for this study were diagnosed with MCC in between the years 1983 and 2013. Causes of death were extracted from the Cause-of-Death Register of Finland by permission from VALVIRA, via personal identity codes (PICs) which are introduced to all citizens and permanent residents of Finland. Patient was alive if he/she was not registered to the Cause-of Death register. Disease stage at diagnosis was determined according to the 8th edition of the American Joint Committee on Cancer (AJCC) cancer staging manual. Formalin-fixed, paraffin-embedded (FFPE) tissue blocks were retrieved from the pathology archives. The primary tumor diagnoses were confirmed by a senior researcher with special expertise in MCC pathology (TB). The criteria for diagnosis of MCC are described in detail elsewhere [15].

Tissue microarray (TMA) blocks were used for IHC. From the FFPE primary tumor samples, representative tumor regions were first defined from hematoxylin & eosin -stained sections and marked. Tissue cores of 0.6-mm in diameter from each tumor sample were inserted into the TMA blocks. 5 μm sections were cut and processed from the TMA blocks for IHC.

### MCPyV detection

The MCPyV status of the tumors was defined based on the immunohistochemical expression of the large T-antigen (LT) which is known to correlate with the presence of MCPyV DNA [27]. The LT expression was detected by using a mouse monoclonal antibody (CM2B4, sc-136172; Santa Cruz Biotechnology Inc.). Binding of the primary antibody was detected by using a PowerVision+ Poly-HRP Histostaining Kit (Immunovision Technologies Co.) according to the manufacturer’s instructions. This process is described in detail elsewhere [27]. A sample was considered MCPyV-positive if LT expression was present.

### Immunohistochemistry

TMA sections were deparaffinized by using a Tissue-Tek® DRS™ instrument (Sakura). Endogenous peroxidase was blocked by immersion in 0,85% H_2_O_2_ (35% Arcos organics, the Netherlands) for 30 minutes at room temperature. ALK Antigen retrieval was performed by heating slides for 10 minutes in a target retrieval solution (EnVision™ FLEX Target Retrieval Solution HIGH pH, Denmark) and for p-ALK 15 min (EnVision™ FLEX Target Retrieval Solution LOW pH, Denmark) using a pressure cooker (decloaking chamber, Biocare Medical). ALK expression was detected using a rabbit monoclonal antibody (clone D5F3®, Cell Signaling Technology; dilution 1:150,) and the presence of p-ALK was detected using a monoclonal rabbit antibody (Tyr1604, Cell Signaling technology; dilution 1:25). Antibodies were diluted in a normal antibody diluent (Normal antibody diluent, ImmunoLogic, the Netherlands). Sections were incubated with the primary antibodies overnight at +4°C. Antibodies were detected using BrightVision polyHRP-Anti-Rabbit IgG (ImmunoLogic, Amsterdam, The Netherlands) and immunostained using Vector laboratories ImmPACT[R] DAB Substrate Kit, Peroxidase (dilution 1 drop per 1 ml) for 5 minutes at room temperature. Finally, slides were counterstained using Mayers Hematoxylin (Lillie´s Modificatin, Dako, USA) and mounted (Eukitt® Quick-hardening mounting medium, SIGMA-ALDRICH®, Germany).

### Interpretation of immunohistochemistry

Slide scanning was provided by BioBank Helsinki using the 3DHISTECH Pannoramic 250. Immunostainings for ALK and p-ALK were analyzed by using the CaseViewer (3DHISTEC, version 2.2). In concordance with previous studies using D5F3® antibody for ALK [11,13], there was a significant variation of staining intensity between individual samples and therefore the staining was scored as either negative, low, intermediate or high. The stainings were scored in a blinded fashion by two researchers (JJ and TB) and cases with conflicting scores were reviewed by the senior pathologist (TB) who made the final scoring.

### MCC cell lines

The MCC cell lines used in this study: MCC13, MCC14_2 and MCC26 [28,29], UISO [30], MKL-1 [31], MKL-2 [32,33], MS1 [34] and PeTa and WaGa [35,36] have been described in detail previously. Five of the cell lines were MCPyV-positive (MS1, PeTa, WaGa, MKL1 and MKL2) and four MCPyV-negative (MCC14_2, UISO, MCC26 and MCC13). MCPyV DNA was detected using real-time PCR as described previously [15]. The UISO cell line was kindly provided by Professor Annamari Ranki (University of Helsinki, Helsinki Finland). PeTa, WaGa and MKL2 were gratefully provided by Professor Roland Houben (University Hospital Würzburg, Würzburg, Germany) and the remaining were obtained from The European Collection of Authenticated Cell Cultures (ECACC) cell culture collection. The NCI-H2228 non-small cell lung cancer cell line that contains the *EML4-ALK* fusion gene and constitutively activated ALK enzyme was included as the positive control in the study. The NCI-H2228 was obtained from the European Collection of Authenticated Cell Culture (ECACC). The MCC lines were grown in RPMI-1640 supplemented with 10% fetal calf serum, 100Uml^−1^ penicillin, and 0.1mgml^−1^ streptomycin. S1 Table provides the information on the origin and *in vitro* features of the nine MCC cell lines used in this study.

### Paraffin-embedded cell blocks

The MCC cell lines were grown up to 70% confluence, detached, counted (~8 million cells/tube), washed twice with PBS (Corning, REF 21-040-CV) prior to splitting the cells into two Eppendorf tubes and centrifuged (10 000 rpm/~9600 g) to form a dense cell pellet. The supernatant was removed, and the cell pellet was resuspended in 60μl human plasma (+4°C). Then, 60 μl of 100 NIH-U/ml thrombin (Merck, REF 1.12374.0001) at +4°C was added to the cell suspension to clot the cells. Next, 1 ml of 5% formalin was added to detach the clots from the Eppendorf tubes, after which the clots were transferred to a larger tube containing ~6 ml of 5% formalin. The cell clots were fixed in 5% formalin for 2 hours at room temperature. Following fixation, the cell clots were subjected to ascending alcohol/xylen series (15 min 70% EtOH, 5 min 94% EtOH, 10 min 94% EtOH, 15 min ABS EtOH, 15 min ABS EtOH, 15 min ABS EtOH, 15 min xylen, 15 min xylen, 15 min xylen) to dehydrate the samples. The cell clots were then immersed in paraffin overnight, forming FFPE-like histological samples. The ALK and p-ALK immunostaining of the cell line samples was graded as negative, low or high based on the percentage of cells expressing. Expression of a given cell line was considered high if over 20% of cells showed expression. In the cell line samples, the staining intensity did not fluctuate and therefore the scoring was based on the percentage of staining cells.

### Statistical analysis

Frequency tables were analyzed using the Pearson’s chi-square test and the Fisher´s Exact test. The Kruskal Wallis and Mann Whitney U tests were used to analyze continuous variables. The cumulative survival was estimated with the Kaplan-Meier method. Survival between groups were calculated using the Mantel-Cox method. Overall and MCC-specific survivals were calculated from the date of the diagnosis until death and until death due to MCC, respectively. Patients that remained living at the end of the follow-up period were censored. Multivariable survival analysis was done by using stepwise Cox regression analysis. All p values are 2-sided, and the statistical analyses were performed using IBM’s SPSS Statistics (Version 25). P values less than 0.05 were considered significant.

## Conclusions

In conclusion, we showed here that in MCC ALK occurs commonly in an active phosphorylated form and the presence of p-ALK correlates with MCPyV positivity and ultimately with longer survival. However, contrary to patient derived samples, in MCC cell lines ALK phosphorylation is scarce and therefore more advanced disease models are necessary in order to resolve whether ALK is a useful treatment target in MCC.

## Supporting information

S1 Table The characterization of the nine MCC cell lines utilized in this study. (DOCX)
