# Supplementary material for: ALK is frequently phosphorylated in Merkel cell carcinoma and associates with longer survival
Source: PLoS One. 2021 May 24;16(5):e0252099. doi: 10.1371/journal.pone.0252099 (PMC8143417; doi:10.1371/journal.pone.0252099)
Supplement: S1 Table — (DOCX) [file pone.0252099.s001.docx]

Supplementary Table 1. Cell line characterisation. Cr-A = chromogranin A, NSE = neuron specific enolase.

| **Merkel cell polyoma virus positivity** | **Cell line** | **Source** | **Neuroendocrine marker expression** | **Morphology** | **References** |
| --- | --- | --- | --- | --- | --- |
|  |  | Sex/Age/ Location | **Classic** - positive for Cr- A and NSE, presence of neurosecretory granules  **Variant** - no neurosecretory granules or express Cr- A, but may or may not express NSE | **Type I:** tight spherical clusters suspended in the medium. **Type II:** as type I, but more loosely arranged cluster, **Type III:** flat 2-D clusters. **Type IV:** adherent monolayer. |  |
| **Positive** | MS1 | F/59 yrs/ tumour biopsy; derived from metastatic site: Adrenal gland. | Classic | III | Guastafierro, A., et al. (2013). Characterization of an early passage Merkel cell polyomaviruspositive Merkel cell carcinoma cell line, MS-1, and its growth in NOD scid gamma mice. J. Virol. Methods 187, 6–14. |
|  | PeTa | M/ 65 yrs/primary tumour, back | Classic | Undefined | Houben R., et al. (2013). Mechanisms of p53 restriction in Merkel cell carcinoma. J Invest Dermatol.133(10):2453-2460 |
|  | WaGa | M/67/ ascites | Classic | Singe cell suspension | Houben, R., et al. (2010). "Merkel cell polyomavirus-infected Merkel cell carcinoma cells require expression of viral T antigens." J Virol 84(14): 7064-7072. |
|  | MKL1 | M /26 yrs. /nodal metastasis | Classic | III | Rosen, S. T., et al. (1987). "Establishment and characterization of a neuroendocrine skin carcinoma cell line." Lab Invest 56(3): 302-312. |
|  | MKL2 | M / 72 yrs. / skin primary tumour | Classic | III | Martin, E. M., et al. (1991). "Parathyroid hormone-related protein, chromogranin A, and calcitonin gene products in the neuroendocrine skin carcinoma cell lines MKL1 and MKL2." Bone Miner 14(2): 113-120 |
| **Negative** | MCC14_2 | M/80 yrs/ nodal metastasis | Variant | IV | Leonard, J. H., et al. (1995). "Characterisation of four Merkel cell carcinoma adherent cell lines." Int J Cancer 60(1): 100-107. |
|  | UISO | F/ 46 yrs. / right anterior thigh, primary tumour | Variant | IV | Ronan, S. G., et al. (1993). "Merkel cell carcinoma: in vitro and in vivo characteristics of a new cell line." J Am Acad Dermatol 29(5 Pt 1): 715-722 |
|  | MCC26 | F / 82 yrs. / tumour recurrence lateral calf | Variant | IV | Van Gele, M., et al. (2002). "Combined karyotyping, CGH and M-FISH analysis allows detailed characterization of unidentified chromosomal rearrangements in Merkel cell carcinoma." Int J Cancer 101(2): 137-145. |
|  | MCC13 | F / 80 yrs. / metastatic cervical node biopsy, primary tumour in the nose | Variant | IV | Leonard, J. H., et al. (1995). "Characterisation of four Merkel cell carcinoma adherent cell lines." Int J Cancer 60(1): 100-107. |
